# Supplementary material for: The RNA-binding protein Puf5 and the HMGB protein Ixr1 contribute to cell cycle progression through the regulation of cell cycle-specific expression of CLB1 in Saccharomyces cerevisiae
Source: PLoS Genet. 2022 Jul 29;18(7):e1010340. doi: 10.1371/journal.pgen.1010340 (PMC9365169; doi:10.1371/journal.pgen.1010340)
Supplement: S1 Table — (DOCX) [file pgen.1010340.s001.docx]

**S1 Table. The strains used in this study.**

| Strain | Genotype | Source |
| --- | --- | --- |
| 10BD | *MATa/MATα ade2/ade2 trp1/trp1 can1/can1 leu2/leu2 his3/his3 ura3/ura3* | 1 |
| 10BD-p5c2 | *MATa/MATα ade2/ade2 trp1/trp1 can1/can1 leu2/leu2 his3/his3 ura3/ura3 PUF5/puf5Δ::CgLEU2 CLB2/clb2Δ::CgHIS3* | This study |
| 10BD-p5c1 | *MATa/MATα ade2/ade2 trp1/trp1 can1/can1 leu2/leu2 his3/his3 ura3/ura3 PUF5/puf5Δ::CgTRP1 CLB1/clb1Δ::CgHIS3* | This study |
| 10BD-p5l1c2c1 | *MATa/MATα ade2/ade2 trp1/trp1 can1/can1 leu2/leu2 his3/his3 ura3/ura3 PUF5/puf5Δ::CgTRP1 LRG1/lrg1Δ::KlURA3 CLB2/clb2Δ::CgHIS3 CLB1/clb1Δ::CgLEU2* | This study |
| 10BD-p5l1c2i1 | *MATa/MATα ade2/ade2 trp1/trp1 can1/can1 leu2/leu2 his3/his3 ura3/ura3 PUF5/puf5Δ::CgTRP1 LRG1/lrg1Δ::KlURA3 CLB2/clb2Δ::CgHIS3 IXR1/IXR1Δ::CgLEU2* | This study |
| 10BD-p5l1c2f1 | *MATa/MATα ade2/ade2 trp1/trp1 can1/can1 leu2/leu2 his3/his3 ura3/ura3 PUF5/puf5Δ::CgTRP1 LRG1/lrg1Δ::KlURA3 CLB2/clb2Δ::CgHIS3 FKH1/fkh1Δ::CgLEU2* | This study |
| 10BD-p5l1c2f2 | *MATa/MATα ade2/ade2 trp1/trp1 can1/can1 leu2/leu2 his3/his3 ura3/ura3 PUF5/puf5Δ::CgTRP1 LRG1/lrg1Δ::KlURA3 CLB2/clb2Δ::CgHIS3 FKH2/fkh2Δ::CgLEU2* | This study |
| 10BD-p5c2hi1 | *MATa/MATα ade2/ade2 trp1/trp1 can1/can1 leu2/leu2 his3/his3 ura3/ura3 PUF5/puf5Δ::CgLEU2 CLB2/clb2Δ::CgHIS3 HIR1/hir1Δ::CgTRP1* | This study |
| 10BD-p5l1c2hf1 | *MATa/MATα ade2/ade2 trp1/trp1 can1/can1 leu2/leu2 his3/his3 ura3/ura3 PUF5/puf5Δ::CgTRP1 LRG1/lrg1Δ::KlURA3 CLB2/clb2Δ::CgHIS3 HFI1/hfi1Δ::CgLEU2* | This study |
| 10BD-p5l1c2s2 | *MATa/MATα ade2/ade2 trp1/trp1 can1/can1 leu2/leu2 his3/his3 ura3/ura3 PUF5/puf5Δ::CgTRP1 LRG1/lrg1Δ::KlURA3 CLB2/clb2Δ::CgHIS3 STE12/ste12Δ::CgLEU2* | This study |
| 10BD-i1f1f2 | *MATa/MATα ade2/ade2 trp1/trp1 can1/can1 leu2/leu2 his3/his3 ura3/ura3 PUF5/puf5Δ::CgTRP1 IXR1/ixr1Δ::CgLEU2 FKH1/fkh1Δ::CgHIS3 FKH2/fkh2Δ::CgHIS3* | This study |
| WT | *MATa ade2 trp1 can1 leu2 his3 ura3* | This study |
| WT | *MATα ade2 trp1 can1 leu2 his3 ura3* | This study |
| *puf5∆* | *MATα ade2 trp1 can1 leu2 his3 ura3 puf5Δ::CgTRP1* | This study |
| *puf5∆* | *MATa ade2 trp1 can1 leu2 his3 ura3 puf5Δ::CgTRP1* | This study |
| *puf5∆* | *MATa ade2 trp1 can1 leu2 his3 ura3 puf5Δ::CgHIS3* | This study |
| *clb2∆* | *MATα ade2 trp1 can1 leu2 his3 ura3 clb2Δ::CgLEU2* | This study |
| *ixr1∆* | *MATα ade2 trp1 can1 leu2 his3 ura3 ixr1Δ::CgLEU2* | This study |
| *bar1∆* | *MATa ade2 trp1 can1 leu2 his3 ura3 bar1∆::CgHIS3* | This study |
| *bar1∆*  *puf5∆* | *MATa ade2 trp1 can1 leu2 his3 ura3 bar1∆::CgHIS3 puf5Δ::CgTRP1* | This study |
| *bar1∆*  *ixr1∆* | *MATa ade2 trp1 can1 leu2 his3 ura3 bar1∆::CgHIS3 puf5Δ::CgTRP1 ixr1Δ::CgLEU2* | This study |
| *bar1∆*  *ccr4∆* | *MATa ade2 trp1 can1 leu2 his3 ura3 bar1∆::CgHIS3 puf5Δ::CgTRP1 ccr4Δ::CgLEU2* | This study |
| *bar1∆*  *puf5∆*  *ixr1∆* | *MATa ade2 trp1 can1 leu2 his3 ura3 bar1∆::CgHIS3 puf5Δ::CgTRP1 ixr1Δ::CgLEU2* | This study |
| *puf5∆ clb2∆* | *MATα ade2 trp1 can1 leu2 his3 ura3 puf5Δ::CgHIS3 clb2Δ::CgLEU2* | This study |
| *puf5∆ ixr1∆* | *MATα ade2 trp1 can1 leu2 his3 ura3 puf5Δ::CgHIS3 ixr1Δ::CgLEU2* | This study |
| *puf5∆ clb2∆ ixr1∆* | *MATα ade2 trp1 can1 leu2 his3 ura3 puf5Δ::CgHIS3 clb2Δ::CgLEU2 ixr1Δ::CgLEU2* | This study |

**References**

1. Tadauchi T, Matsumoto K, Herskowitz I, Irie K. Post-transcriptional regulation through the *HO* 3’-UTR by Mpt5, a yeast homolog of Pumilio and FBF. EMBO J. 2001; 20: 552–561. doi.org/10.1093/emboj/20.3.552
