# Supplementary material for: The RNA-binding protein Puf5 and the HMGB protein Ixr1 contribute to cell cycle progression through the regulation of cell cycle-specific expression of CLB1 in Saccharomyces cerevisiae
Source: PLoS Genet. 2022 Jul 29;18(7):e1010340. doi: 10.1371/journal.pgen.1010340 (PMC9365169; doi:10.1371/journal.pgen.1010340)
Supplement: S2 Table — (DOCX) [file pgen.1010340.s002.docx]

**S2 Table. Plasmids used in this study**

| Plasmids | Relevant markers | Reference |
| --- | --- | --- |
| YCplac33 | *URA3, CEN-ARS* | 1 |
| YEplac195 | *URA3* | 1 |
| YEp13 | *LEU2* | 1 |
| YEp13-PUF5 | *LEU2, PUF5* | This study |
| YEplac195-PUF5 | *URA3, PUF5* | This study |
| YEplac195-CLB2 | *URA3, CLB2* | This study |
| YEplac195-CLB1 | *URA3, CLB1* | This study |
| YCplac33-IXR1 | *URA3, CEN-ARS, IXR1* | This study |
| YCplac33- CLB1-3HA-CLB1 3’-UTR | *URA3, CEN-ARS, CLB1-3HA* | This study |
| YCplac33- IXR1-3HA-IXR1 3’-UTR | *URA3, CEN-ARS, IXR1-3HA* | This study |
| YCplac33- IXR1-3HA-ADH1 3’-UTR | *URA3, CEN-ARS, IXR1-3HA* | This study |
| YCplac33- CLB1-GFP-ADH1 3’-UTR | *URA3, CEN-ARS, GFP* | This study |
| YCplac33- CLB2-GFP-ADH1 3’-UTR | *URA3, CEN-ARS, GFP* | This study |
| YCplac33- MCM2-GFP-IXR1 3’-UTR | *URA3, CEN-ARS, GFP* | This study |
| pCgLEU2 | *C. glabrata LEU2* in pUC19 | 2 |
| pCgHIS3 | *C. glabrata HIS3* in pUC19 | 2 |
| pCgTRP1 | *C. glabrata TRP1* in pUC19 | 2 |
| pKlURA3 | *K. lactis URA3* in pUC19 | 2 |
| pFA6a-3HA-kanMX6 | *3HA-ADH1* terminator, kanamycin resistance cassette | 3 |

**References**

1. Gietz RD, Sugino A. New yeast-Escherichia coli shuttle vectors constructed with in vitro mutagenized yeast genes lacking six-base pair restriction sites. *Gene*. 1988;74(2):527-534. doi:10.1016/0378-1119(88)90185-0

2. Sakumoto N, Mukai Y, Uchida K, Kouchi T, Kuwajima J, Nakagawa Y, et al. A series of protein phosphatase gene disruptants in *Saccharomyces cerevisiae*. Yeast. 1999; 15: 1669-1679. doi:10.1002/(SICI)1097-0061(199911)15:15<1669::AID-YEA480>3.0.CO;2-6

3. Longtine MS, Mckenzie III A, Demarini DJ, Shah NG, Wach A, Brachat A, et al. Additional modules for versatile and economical PCR-based gene deletion and modification in *Saccharomyces cerevisiae*. Yeast. 1998; 14: 953–961. doi:10.1002/(SICI)1097-0061(199807)14:10<953::AID-YEA293>3.0.CO;2-U
