# Supplementary material for: The RNA-binding protein Puf5 and the HMGB protein Ixr1 contribute to cell cycle progression through the regulation of cell cycle-specific expression of CLB1 in Saccharomyces cerevisiae
Source: PLoS Genet. 2022 Jul 29;18(7):e1010340. doi: 10.1371/journal.pgen.1010340 (PMC9365169; doi:10.1371/journal.pgen.1010340)
Supplement: S3 Table — (DOCX) [file pgen.1010340.s003.docx]

**S3 Table. Primers used for the gene deletion.**

| Gene | Forward primer | Reverse primer |
| --- | --- | --- |
| *PUF5* | TTCTACGCAAATTTATAAATCAATTACGATTTTTCCAGTTTCTCTTCACAGGAAACAGCTATGACC | AATATTTGTACAGTAAGAAGGAAAGAAAAAGAAAGAAAAAAAAGTAGTTGTAAAACGACGGCCAGT |
| *CLB2* | AAGCCTTTTATTGATTACCCCCTCTCTCTCTTCATTGATCTTATAGCACAGGAAACAGCTATGACC | ATTTATCGATTATCGTTTTAGATATTTTAAGCATCTGCCCCTCTTCGTTGTAAAACGACGGCCAGT |
| *CLB1* | TTCGTCCGTTATATCAACCATCAAAGGAAGCTTTAATCTTCTCATACACAGGAAACAGCTATGACC | TAAAGTAAGGAAGTGAGATTTTGGTTTTCTGTGTAGGCTAGCACCTGTTGTAAAACGACGGCCAGT |
| *IXR1* | TCCATTCTGTGATATACGTACGACGCTAACAGTACCCACAACTGCACACAGGAAACAGCTATGACC | TTTGCGTGGGATAATGTTACAGTGGAAAACTAAAGTTGTTTATTTGGTTGTAAAACGACGGCCAGT |
| *BAR1* | CCTAAAATCATACCAAAATAAAAAGAGTGTCTAGAAGGGTCATATACACAGGAAACAGCTATGACC | TATATTTGATATTTATATGCTATAAAGAAATTGTACTCCAGATTTCGTTGTAAAACGACGGCCAGT |
| *LRG1* | AAAAAGAGCAGACAAATTATCAAACAACAAGTACCGGAGGTGAGCACACAGGAAACAGCTATGACC | GAGAAGAAAAAAAGGAAAATGAGGGGAAACTTACAGTTTCTGCCTAGTTGTAAAACGACGGCCAGT |
| *FKH1* | GCGTTCAATTAGCAAAGAAAGGCTTGGAGAGACACAGTAATAATAACACAGGAAACAGCTATGACC | GTTCTTTATTGTTTAATAATACATATGGGTTCGACGACGCTGAATTGTTGTAAAACGACGGCCAG |
| *FKH2* | GTGCTCCCTCCGTTTCCTTTATTGAAACTTTATCAATGCGCAAGAACACAGGAAACAGCTATGACC | TTCTCATTCATTTCTTTAGTCTTAGTGATTCACCTTGTTTCTTGTCGTTGTAAAACGACGGCCAGT |
| *HFI1* | TAAAAATATAGGGAAAACAAGCCCAGTAGTTTTGATTTCTTCTATCCACAGGAAACAGCTATGACC | CTTTCAATATAATTACAACATACCGCATACACACACTTTTTATACAGTTGTAAAACGACGGCCAGT |
| *HIR1* | CAACGAAGCATAATAAAATTGCCAGTAACCAAAGGTCTCTGATAACCACAGGAAACAGCTATGACC | TAATTATGAGGGAAAAAACTTGTCCAAAGGAAGGGGTATAAGCTTAGTTGTAAAACGACGGCCAGT |
| *STE12* | TATAGCGGAACCGCTTTCTTTATTTGAATTGTCTTGTTCACCAAGGCACAGGAAACAGCTATGACC | CCCGCATTTTTAATTCTTGTATCATAAATTCAAAAATTATATTATAGTTGTAAAACGACGGCCAGT |
