# Supplementary material for: The RNA-binding protein Puf5 and the HMGB protein Ixr1 contribute to cell cycle progression through the regulation of cell cycle-specific expression of CLB1 in Saccharomyces cerevisiae
Source: PLoS Genet. 2022 Jul 29;18(7):e1010340. doi: 10.1371/journal.pgen.1010340 (PMC9365169; doi:10.1371/journal.pgen.1010340)
Supplement: S4 Table — (DOCX) [file pgen.1010340.s004.docx]

**S4 Table. Primers used for the qRT-PCR.**

| Gene | Forward primer | Reverse primer |
| --- | --- | --- |
| *SCR1* | AACCGTCTTTCCTCCGTCGTAA | CTACCTTGCCGCACCAGACA |
| *ACT1* | TGCCGAAAGAATGCAAAAGG | TCTGGAGGAGCAATGATCTTGA |
| *GFP* | CACTGGAGTTGTCCCAATTCTTG | TCCGTATGTTGCATCACCTTCA |
| *CLB1* | TTCCGAGCAAGAAAAGCAGC | TCGTACTCCTCCAGAACCTC |
| *CLB2* | GCCGATGACTTCACCTCCTC | CTGCTGCTTTTCTTGCTCGG |
| *CLB6* | GCGATCAACCTGCTAGATCG | ACGAATAGCTCAGCCTTCCT |
| *ADH1* | TGTCTGTCACACTGACTTGCAC | CACCGATCTTCCAGCCCTTAAC |
| *IXR1* | ACGCGCTACCTCAGGATTTC | CCCGTGACAGAAGGGTTTGT |
| *RNR1* | CCATGGCACCAATGCCTACT | CACCGGATAAGACACGACGG |
| *SIC1* | GGCTTACGTCTCCTCAACGC | CGACCCAATGGTTCCTGCTC |
| *TOS1* | ACTGCCGGTTCTGGTGTCTG | ACCAAGAGCTTGAGCGGAGG |
| *SUN4* | TTCTGTCGAAGATGCTTGTGTTTG | CGTTACCGTTGTTTGGGTTGG |
| *CLB1-3HA* | GAGCGGATCCCCGGGTTAAT | TAGTCCGGGACGTCATACGG |
| *IXR1-3HA* | AGCCGCTCCAACAACAACAA | ACGGATAGCCCGCATAGTCA |
| *PUF5* | ACGGAAGGTGCAAACTGGGA | AGCGGTATTCATGGCAGGCA |
| *AFT1* | GGTCGTGCAAGTGCAAGTGG | CCGTCGGCGATGTTGAGGTA |
| *CAD1* | AGCCTTCCTCGATGATCTGC | AACGGCACCTGTTCGATTTC |
| *FKH1* | AAGGGCTTGTTAATGCGGTG | GGGCACAGCATTCAAGTTCA |
| *FKH2* | CAAGAACATGGCGACGGAAA | TATGTGCAATTCCCAACCGC |
| *HFI1* | TTATCCAGCTGCTTCGCCAG | AGGCTCCGCAGTTTCATTGT |
| *HIR1* | AATGCGGGTGGTAAGGGTTC | CTGGGTTAGCTTGGCGTTCA |
| *HIR3* | TTGCTGGCTCGCATCTTCAA | TGTCGCCCCAACAAAAGTGA |
| *NDT80* | CGACGACGACACGGAAATCA | GGAAGGCACCAACGGACATA |
| *SFP1* | AACTGCGTCGTCCACAACTG | TCGTTGAAACGGCATCGACT |
| *SPT10* | CGCTGAATGATGTGGAGGGT | TGTTGCGGGACTATTCGTGG |
| *SPT20* | GTGGGATCTACCATGGGTAACG | TGTTTGCACTTAAAGCCTGCTG |
| *SPT3* | ACACGACAAGGCCAAAGTCA | GCTACACCGGCACTAGCATC |
| *SRB2* | TGCGACTCGTCAACCTCTTC | TCGATGCCTGCAATCTTGGT |
| *STE12* | AGTTGTTTGCGGATGCGTTG | CGGGCTCATTAACGGGCTT |
| *YAP1* | ACACACCAAACTCCTCCACT | TGGGTTTCTTGGGAATGGGA |
