# Supplementary material for: The RNA-binding protein Puf5 and the HMGB protein Ixr1 contribute to cell cycle progression through the regulation of cell cycle-specific expression of CLB1 in Saccharomyces cerevisiae
Source: PLoS Genet. 2022 Jul 29;18(7):e1010340. doi: 10.1371/journal.pgen.1010340 (PMC9365169; doi:10.1371/journal.pgen.1010340)
Supplement: S5 Table — (DOCX) [file pgen.1010340.s005.docx]

**S5 Table. Fold change of the mRNA level of regulators of *CLB1*.**

The mRNA levels of each gene in wild-type and the *puf5∆* mutant strains harboring the pRS316-3xFLAG-*LRG1* plasmid are presented. The mRNA levels were quantified by qRT-PCR analysis, and the relative mRNA levels were calculated using the *ACT1* reference gene. The data show the fold change of mRNA level relative to the mRNA level in wild-type.

| GENE | wild-type | *puf5Δ* |
| --- | --- | --- |
| *AFT1* | 1 | 1.5 |
| *CAD1* | 1 | 1.4 |
| *FKH1* | 1 | 2.4 |
| *FKH2* | 1 | 1.8 |
| *HFI1* | 1 | 2.4 |
| *HIR1* | 1 | 1.8 |
| *HIR3* | 1 | 1.3 |
| *IXR1* | 1 | 2.0 |
| *NDT80* | 1 | 1.5 |
| *SFP1* | 1 | 1.2 |
| *SPT10* | 1 | 1.4 |
| *SPT20* | 1 | 1.3 |
| *SPT3* | 1 | 1.0 |
| *SRB2* | 1 | 1.1 |
| *STE12* | 1 | 1.6 |
| *YAP1* | 1 | 1.2 |
