# Supplementary figures and images for: The RNA-binding protein Puf5 and the HMGB protein Ixr1 contribute to cell cycle progression through the regulation of cell cycle-specific expression of CLB1 in Saccharomyces cerevisiae
Source: PLoS Genet. 2022 Jul 29;18(7):e1010340. doi: 10.1371/journal.pgen.1010340 (PMC9365169; doi:10.1371/journal.pgen.1010340)

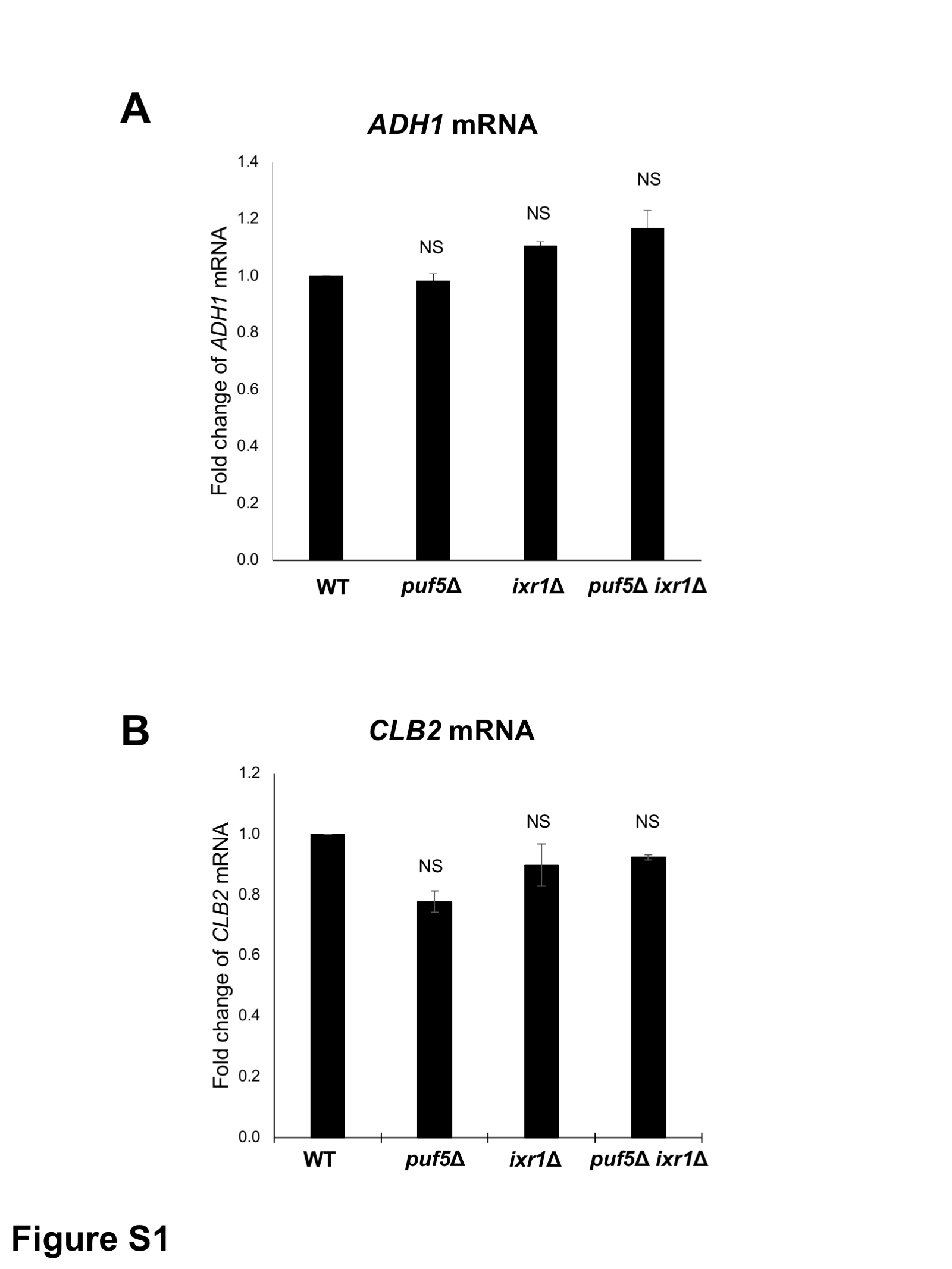

Supplement: S1 Fig — (A) The mRNA levels of ADH1 in wild-type, the puf5Δ mutant, the ixr1Δ mutant, and the puf5Δ ixr1Δ double mutant. The cells were cultured in a YPD medium at 28°C until the exponential phase. The ADH1 mRNA levels were quantified by qRT-PCR analysis, and the relative mRNA levels were calculated using the ACT1 reference gene. The data shows the mean ± SE (n = 3) of the fold change of ADH1 mRNA relative to the mRNA level in wild-type. *P < 0.05, **P < 0.01 as determined by Tukey’s test. (B) The mRNA levels of CLB2 in wild-type, the puf5Δ mutant, the ixr1Δ mutant, and the puf5Δ ixr1Δ double mutant. The cells were cultured in a YPD medium at 28°C until the exponential phase. The CLB2 mRNA levels were quantified by qRT-PCR analysis, and the relative mRNA levels were calculated using the SCR1 reference gene. The data shows the mean ± SE (n = 3) of the fold change of CLB2 mRNA relative to the mRNA level in wild-type. *P < 0.05, **P < 0.01 as determined by Tukey’s test. (TIFF) [file pgen.1010340.s007.tiff]

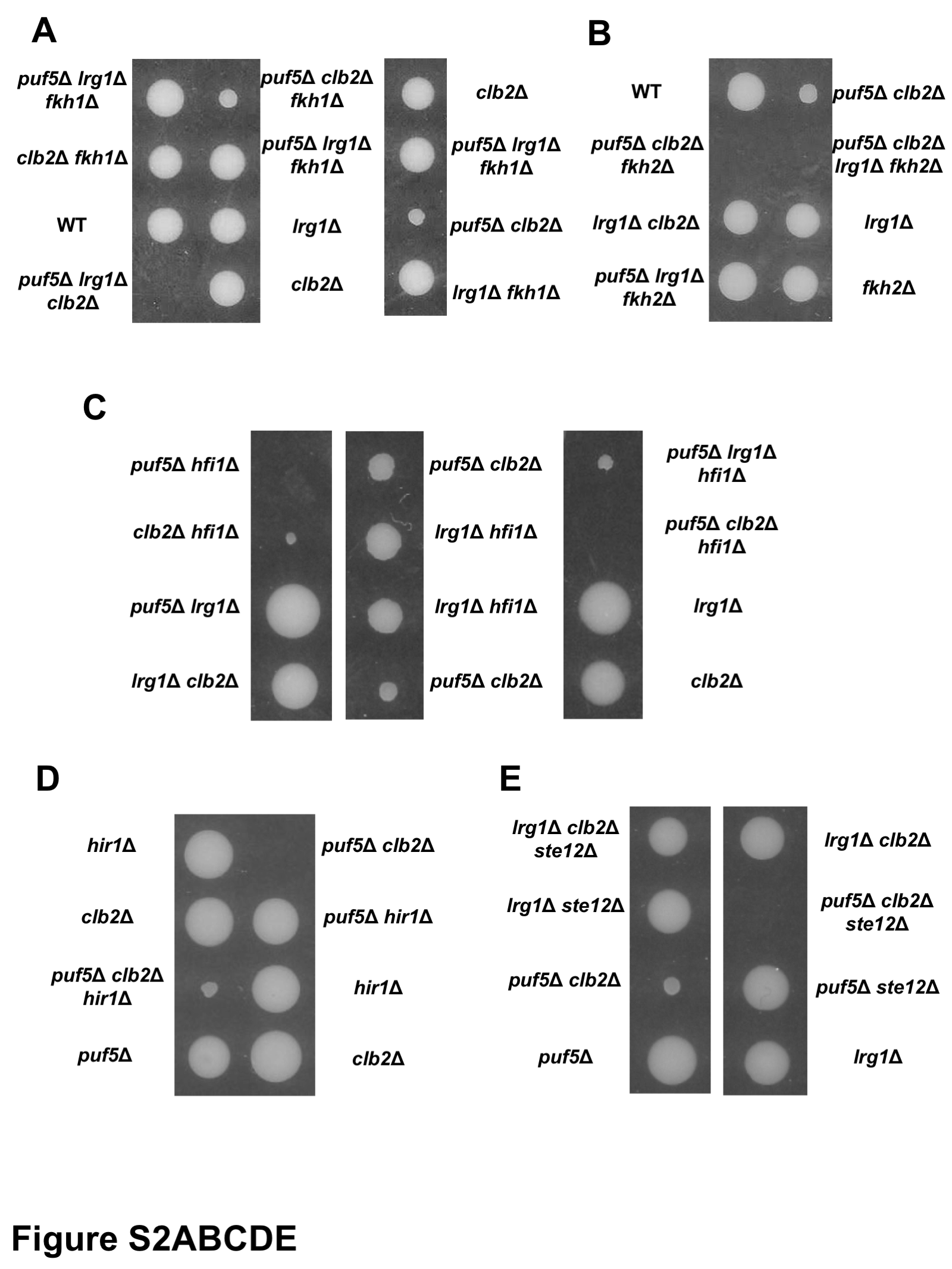

Supplement: S2 Fig — (A) The tetrad analysis of the strains that are heterozygous for the alleles of PUF5, CLB2, FKH1, and LRG1 (A), PUF5, CLB2, FKH2, and LRG1 (B), PUF5, CLB2, HFI1, and LRG1 (C), PUF5, CLB2, and HIR1 (D), PUF5, CLB2, STE12, and LRG1 (E). The cells were sporulated, dissected on a YPD plate containing 10% sorbitol, and cultured at 30°C for 3 days. (TIFF) [file pgen.1010340.s008.tiff]

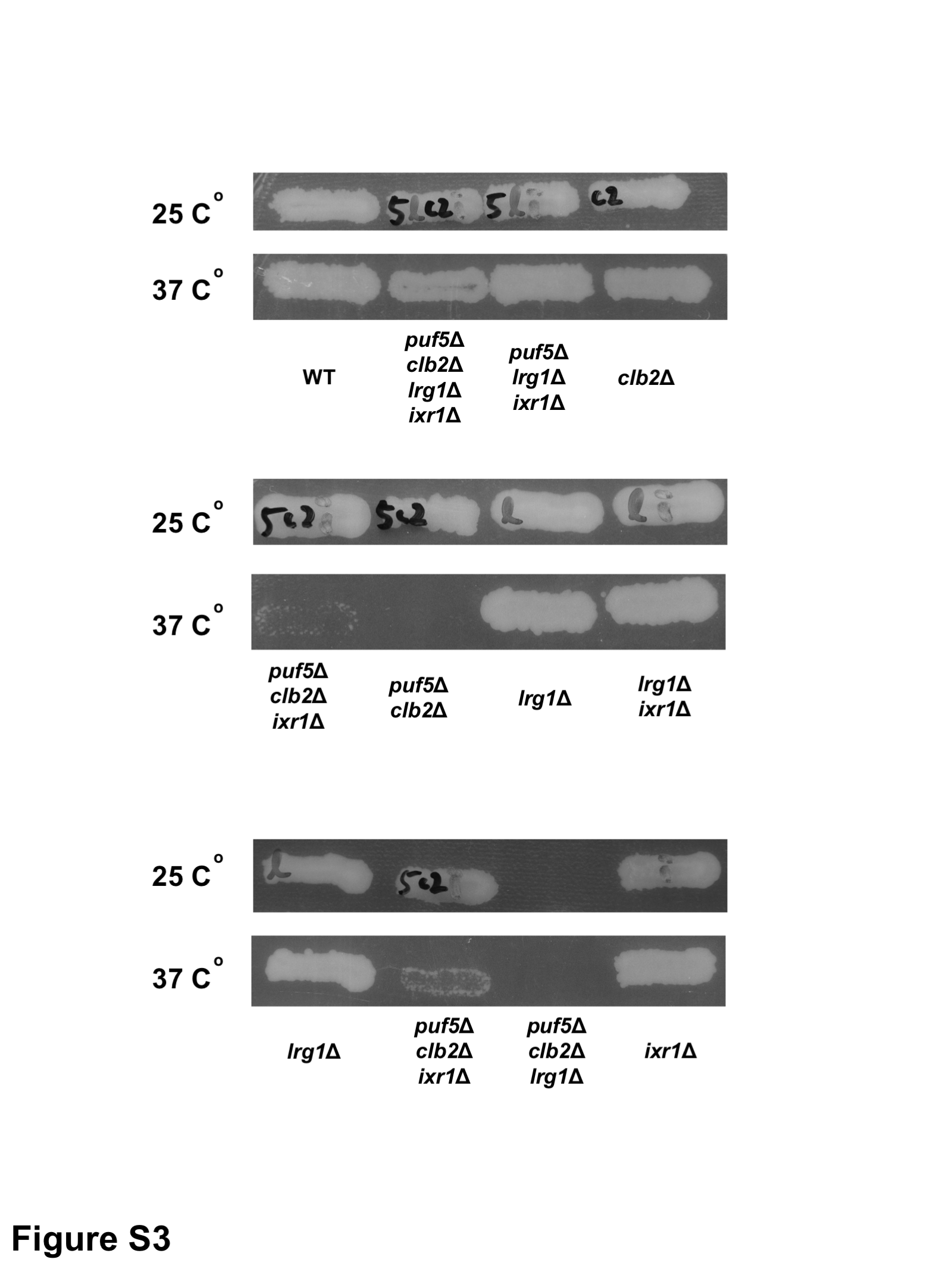

Supplement: S3 Fig — The effect of the ixr1Δ mutation and the lrg1Δ mutation on cell growth at 37°C. The sets of the strains obtained from the tetrad analysis shown in Fig 5B were picked on a YPD plate containing 10% sorbitol and incubated at 25°C for 1 day. Then, the plate was replicated to a YPD plate and incubated at 37°C for 1 day. (TIFF) [file pgen.1010340.s009.tiff]

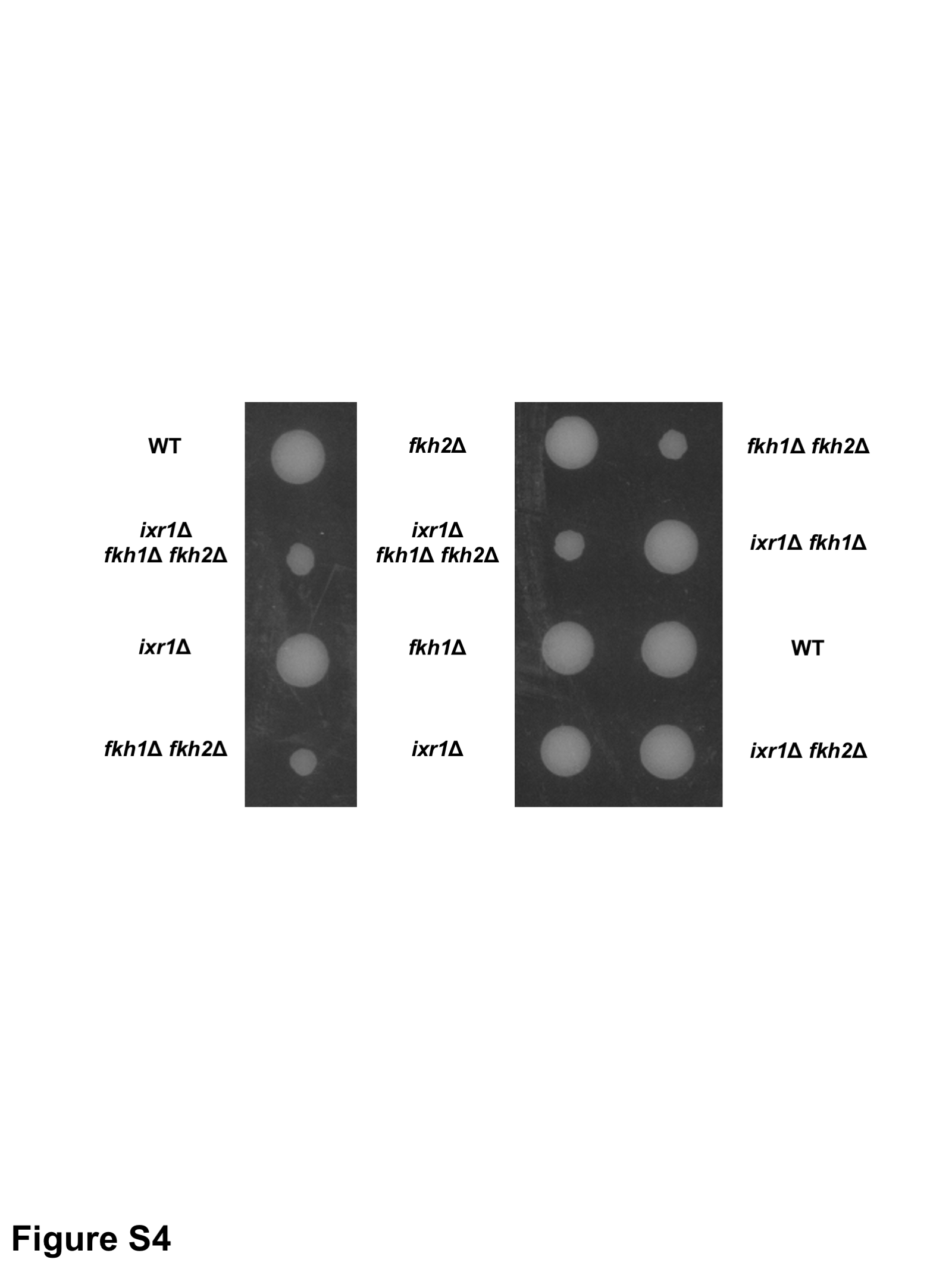

Supplement: S4 Fig — The tetrad analysis of the strains that are heterozygous for the alleles of IXR1, FKH1, and FKH2. The cells were sporulated, dissected on a YPD plate containing 10% sorbitol, and cultured at 30°C for 3 days. (TIFF) [file pgen.1010340.s010.tiff]

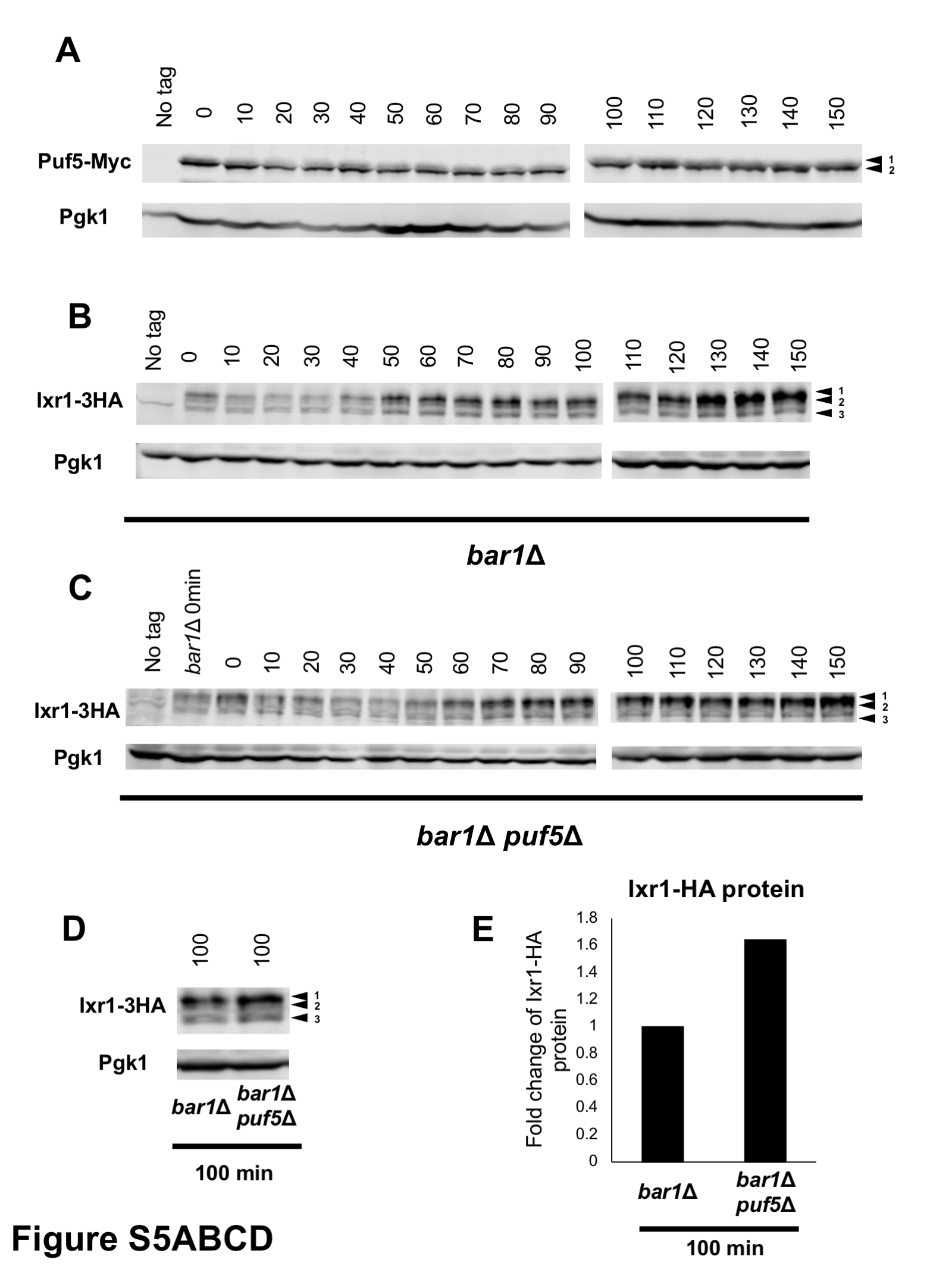

Supplement: S5 Fig — (A) The Puf5 protein level in the synchronized bar1Δ cell integrated PUF5-Myc-ADH1 3´ UTR gene. No tag sample was loaded as a negative control. The arrowheads show two different bands of Puf5-Myc protein observed. Band 1 and band 2 were quantified and normalized with the Pgk1 protein level. Fold change is presented in Fig 12B. (B, C) The Ixr1 protein level in the synchronized bar1Δ cell (B) and bar1Δ puf5Δ mutant (C) harboring the YCplac33-IXR1-HA-IXR1 3´ UTR plasmid. No tag sample was loaded as a negative control. The arrowheads show three different bands of Ixr1-HA protein observed. Band 1 and band 2 were quantified and normalized with the Pgk1 protein level. Fold change is presented in Fig 13C. (D, E) The comparison of the Ixr1 protein level (D) and quantitative analysis data of Ixr1-HA protein (E) between the synchronized bar1Δ cell and bar1Δ puf5Δ mutant harboring the YCplac33-IXR1-HA-IXR1 3´ UTR plasmid. 100 min samples of each strain were loaded. The arrowheads show three different bands of Ixr1-HA protein observed. Band 1 and band 2 were quantified and normalized with the Pgk1 protein level. The data shows the fold change of Ixr1-HA protein relative to the protein level in the bar1Δ cell (E). (TIFF) [file pgen.1010340.s011.tiff]
