# Supplementary material for: The RNA-binding protein Puf5 and the HMGB protein Ixr1 contribute to cell cycle progression through the regulation of cell cycle-specific expression of CLB1 in Saccharomyces cerevisiae
Source: PLoS Genet. 2022 Jul 29;18(7):e1010340. doi: 10.1371/journal.pgen.1010340 (PMC9365169; doi:10.1371/journal.pgen.1010340)
Supplement: Attachment — Submitted filename: Comments_PGENETICS-D-22-00414.docx [file pgen.1010340.s012.docx]

The manuscript entitled "The RNA-binding protein Puf5 and the HMGB protein Ixr1 contribute ..." by Sato et al. describes identification of a key biological process whose failure causes temperature sensitivity in the *puf5∆* strain in *S. cerevisiae*. They started from genetic analysis to identify *CLB2*, one of the B-type cyclin genes, as a multicopy suppressor of the *puf5∆* mutant, and this led to their revealing repression of *CLB1* mRNA, another B-type cyclin, in *puf5∆*. The authors finally reached the finding that Ixr1, a High Mobility Group box B protein, acting as a transcription repressor of *CLB1*, is under the post-transcriptional control of Puf5 through the 3'-UTR of *IXR1* mRNA. They also pointed that Puf5 positively regulates Clb1 expression in an Ixr1-independent manner probably in parallel at the translational level. In addition, they analyzed effects of Puf5 on cell cycle-dependent regulation of *CLB1* mRNA using synchronous cultures.

Strong points)

Using multistep genetical analyses and appropriate reporter constructs, the authors revealed the whole pathway of Clb1 regulation by the Puf5-Ixr1 system and defined a regulation level (transcription, mRNA degradation, and/or translation) of each regulatory step.

Weak points)

The authors analyzed cell-cycle dependent change of *CLB1* expression using synchronous cultures. They claimed that the peak of *CLB1* mRNA expression was not observed in the *bar1∆ puf5∆* mutant. However, the *CLB1* mRNA level in this mutant apparently showed a low and broad peak around 80–100 min after alpha-factor release in every case (Figs. 8C, 9B, and 10B). If taking some elongated cell-cycle of the *bar1∆ puf5∆* cells expected from level changes of *RNR1* and *SIC1* mRNAs into account, this low, broad and delayed peak seems to be a real peak of *CLB1* mRNA according to cell-cycle progression. Thus, the effect of Puf5, whose deletion causes higher expression of Ixr1, can be interpreted as control of the maximum expression level but not cyclic regulation itself. From the mRNA expression change in Figs. 8–10, one complete cell cycle may not have been covered under these experimental conditions (120 min duration) in the case of the *bar1∆ puf5∆* mutant, so that decrease of the *CLB1* mRNA level upon entering a new G1 phase were not clearly observed in the data. The reviewer recommends to clarify whether Ixr1 affects expression maximum of *CLB1* mRNA or its rhythmicity according to cell-cycle progression.

In a similar line, the authors do not seem to care about difference of the cell-cycle phase (e. g. p. 16, lines 399-401), but both *puf5∆* and *ixr1∆* mutations clearly altered cell-cycle progression, especially recovery from alpha-factor arrest; *puf5∆* showed delayed recovery while *ixr1∆* does earlier recovery. Such difference should be precisely described and discussed.

Finally, if Ixr1 affects cell-cycle dependent rhythmicity of the *CLB1* mRNA level, translational regulation of *IXR1* mRNA may also be important (see Fig. 7D). Did the authors examine cyclic expression of Ixr1 protein and the effect of *puf5* deletion on it?

Minor comments

1) The authors compared growth phenotypes of several combinations of gene deletions by tetrad analysis in Fig. 3D. But their tetrad sets failed to include an important "positive control" of defective growth, namely a *puf5∆ clb2∆* double mutant. Since the PLOS Genetics data policy does not allow "data not shown," appropriate tetrad sets including *puf5∆ clb2∆* should replace the current tetrad data.

2) In Figs. 8–10, visibility of mutant plots is quite poor. The reviewer recommends to use color lines or black dash lines to distinguish data of mutant strains from those of the control. In addition, the authors should clearly indicate what value is set to 1 in the ordinate of the graphs.
