# Supplementary material for: The RNA-binding protein Puf5 and the HMGB protein Ixr1 contribute to cell cycle progression through the regulation of cell cycle-specific expression of CLB1 in Saccharomyces cerevisiae
Source: PLoS Genet. 2022 Jul 29;18(7):e1010340. doi: 10.1371/journal.pgen.1010340 (PMC9365169; doi:10.1371/journal.pgen.1010340)
Supplement: Attachment — Submitted filename: Sato_response-to-reviewers_20220621-2.docx [file pgen.1010340.s013.docx]

**Our responses to the reviewers’ comments and changes in the revised manuscript**

Tittle: The RNA-binding protein Puf5 and the HMGB protein Ixr1 contribute to cell cycle progression through the regulation of cell cycle-specific expression of *CLB1* in Saccharomyces cerevisiae

Authors: Megumi Sato, Kaoru Irie, Yasuyuki Suda, Tomoaki Mizuno, Kenji Irie

**Reviewer #1**

1. Because authors use the ADH1 3’ UTR as a control, it would be informative to show the expression of ADH1 in all different mutants as a supplemental figure to show that its regulation is not altered in different mutants.

According to the reviewer’s comment, we added the data presenting *ADH1* mRNA levels in wild-type, the *puf5*Δ mutant, the *ixr1*Δ mutant, and the *puf5*Δ *ixr1*Δ double mutant in Supplementary Figure 1A of the revised manuscript.

2. The authors cite reference #7 for the interaction between IXR1 and Puf5. Does Puf5 also bind to any CLB or CLN mRNAs?

Miller et al. (2018) reported that Puf5 binds to *CLB1* mRNA, *CLB2* mRNA, and *CLN1* mRNA (Miller et al., 2018. Genome-Wide Mapping of Decay Factor-mRNA Interactions in Yeast Identifies Nutrient-Responsive Transcripts as Targets of the Deadenylase Ccr4.). Lapointe et al. (2017) also reported that Puf5 binds to *CLN3* mRNA (Lapointe et al., 2017. Architecture and dynamics of overlapped RNA regulatory networks.).

Although the binding of Puf5 to the four cyclin mRNAs has been reported in these previous manuscripts, our microarray data showed that the only *CLB1* mRNA level was increased in the *puf5*Δ mutant. We discussed about the binding of Puf5 to *CLN* and *CLB* mRNAs in the discussion section in the revised manuscript (page 25, line 598-602)

3. The authors show that the cell-cycle-dependent expression of some genes is dependent on Puf5 and Ixr1. Does the expression of PUF5 mRNA or Puf5 protein change throughout the cell cycle?

According to the reviewer’s comment, we examined the expression of *PUF5* mRNA and Puf5 protein throughout the cell cycle. We added the results in Figure 12 and supplementary Figure 5A of the revised manuscript.

4. Line 48: RNA binding proteins bind to both 5’ and 3’ UTRs, not only 3’ UTRs.

We edited the sentences in the Introduction section of the revised manuscript (page 4, line 58-61).

5. The authors should explain the genomic DNA library preparation and how they performed the screening in the methods section.

We added the citation of the genomic DNA library and describe the screening methods more precisely in the Materials and Methods section of the revised manuscript (page 8, line 160-173).

6. Line 211: “CLB1 suppressed the temperature sensitivity phenotype”. This seems to be only true at 35˚C and not 37˚C. Authors could comment more on this and maybe mention it in the discussion.

According to the reviewer’s comment, we described the data is the results section of the revised manuscript (page 12, line 256-259) and discussed about the weak suppression of the temperature-sensitiveness by *CLB2* in the discussion section of the revised manuscript (page 26, line 633 to page 27, line 657).

7. Line 289: Typo, should read as “promoter”.

We thoroughly edited the sentences of the revised manuscript (Page 15, line 349).

8. Typo, Figure 7D y-axis label should read as “Fold”.

We thoroughly edited the label on the Figure 7D of the revised manuscript.

9. “expressions” is used several times interchangeably with “expression”. It should always be singular.

We thoroughly edited the use of the “expressions” and “expression”, and unified into “expression” in the revised manuscript.

10. Line 347: Fig 7C does not show the correct information. This should refer to Fig 6D.

We thoroughly edited the data label of the revised manuscript (Page 18, line 434).

11. Overall, authors should revise their discussion to reflect how their work fits into the field of RNA binding proteins and cell cycle regulation.

According to the reviewer’s comment, we additionally discussed about the insights into the cell cycle regulation and RNA regulatory network that our work gives in the discussion section of the revised manuscript (page 28, line 683 to page 30, line 726).

**Reviewer #2**

Major points

1. The screening procedure is explained very minimally, e.g. how was the screening library constructed (or cite relevant paper). Why was Lrg1 co-transformed with the screening library? For a better understanding, it would be good to add a description in the results section and to expand the M&M with details of the library. Were SSD1, CLB2 and ZDS1 the only hits in the screen. It would be nice to include a table as part of the supplements with all the hits (even though they might not be validated) for future reference.

According to the reviewer’s comment, we added the citation of the genome library in the revised manuscript. We co-transformed the pRS316-3xFLAG-LRG1 to obtain not only suppressor of the *puf5*Δ mutant but also negative regulator of *LRG1*. From this screening, we obtained two hits of *SSD1*, one hit of *CLB2*, and one hit of *ZDS1*. Other suppressors could not be acquired. We described the screening procedure particularly in the Material and Methods section of the revised manuscript (page 8, line 160-173).

2. Figure 4 examines a potential role in transcriptional regulation of Clb1 mRNA. As PUF5 has been implicated to impact mRNA stability, this result in itself is not convincing enough to rule out any post-transcriptional roles of PUF5 in this experiment. The authors should provide additional controls, e.g. using the ADH1 promoter in the same fashion.

According to the reviewer’s comment, we examined the expression of the *GFP* reporter construct, *CLB2* promoter-*GFP*-*ADH1* 3´ UTR in wild-type and the *puf5*Δ mutant. We also examined the expression in the *ixr1*Δ mutant and the *puf5*Δ *ixr1*Δ double mutant. We utilized the *CLB2* promoter, because the endogenous *CLB2* mRNA levels were invariable among the mutants. We also confirmed that the endogenous *ADH1* mRNA levels were not affected by the *puf5∆* or *ixr1∆* mutation. These results were added in Figure 4B, 6F and Supplementary Figure 1A, B of the revised manuscript.

3. To identify potential regulators of Clb1, the authors screened SGD and selected 16 potential regulators (Fig. 5A) and claim that IXR1, FKH1 and FKH2 increase expression in a puf5D mutant, but do not show the corresponding data. This must be included in the manuscript (for all regulators tested). Same holds true for the subsequent tetrad analysis of the fkh1/2 deletion (line 316/317).

According to the reviewer’s comment, we added the results of the screening of the possible regulators of *CLB1*. In addition to *IXR1*, *FKH1* and *FKH2*, *HFI1*, *HIR1*, and *STE12* also showed a certain increase in the mRNA levels in the *puf5*Δ mutant. We have performed the tetrad analysis of all the deletions. We added the results of the mRNA expression of the genes in S5 Table and the tetrad analysis data in Supplementary Figure 2 of the revised manuscript.

4. Fig. 5E should have the full set of controls, including *puf5*Δ, *clb2*Δ and *puf5*Δ *clb2*Δ cells to directly compare the phenotypes.

According to the reviewer’s comment, we included the image of the morphology of wild-type, the *puf5*Δ mutant, the *clb2*Δ mutant, and the *puf5*Δ *clb2*Δ double mutant cells in Figure 5E of the revised manuscript.

5. To show more directly that Ixr1 is indeed a target of Puf5, the authors should consider to perform a Puf5 RIP and analyze enrichments of Ixr1 mRNA. More important would be to directly show that Ixr1 regulates *CLB1* transcription and that Ixr1 is indeed recruited to *CLB1* promoter (with increased levels in *puf5*Δ).

According to the reviewer’s comment, we performed the Puf5 RIP analysis to clarify the interaction of Puf5 and *IXR1* mRNA. Consequently, we could confirm the binding of Puf5 to the *IXR1* mRNA. We also examined the affect of the Puf5-binding element within the 3’ UTR of *IXR1* mRNA on the Puf5-binding and the Puf5-mediated regulation. We added these results in Figure 7E-G and Figure 8 in the revised manuscript.

As for the interaction between Ixr1 protein and the *CLB1* promoter, previous genome-wide ChIP analysis has not reported the binding of Ixr1 to the *CLB1* promoter (Vizoso-Vázquez et al., 2018. Ixr1 Regulates Ribosomal Gene Transcription and Yeast Response to Cisplatin.). Therefore, we considered a possibility that Ixr1 indirectly regulates the *CLB1* expression. Since Fkh1 and Fkh2 are known to positively regulate *CLB1* expression, we examined the genetic interaction between *IXR1* and *FKH1*/*FKH2* by tetrad analysis. We added these results in the Supplementary Figure 4 of the revised manuscript. We also discussed the machinery of the Ixr1 function in the discussion section of the revised manuscript (page 25, line 607 to page 26, line 617).

Minor points

1. Tetrad dissection was performed at 30oC according the figure legend. However, in the text, it is written in a way that suggests 25oC (line 217), which renders Fig. 1D a bit confusing.

We thoroughly edited the description in the results section of the revised manuscript (page 12, line 268).

2. As the effect of *puf5*Δ appears to be stronger on the level of protein compared to RNA, it would be good to include a western blot of Clb1-HA expression upon over expression of PUF5.

According to the reviewer’s comment, we examined the effect of YEp-*PUF5* on the Clb1-HA protein level. For the observation of the Clb1-HA protein level, we used the YCplac33-*CLB1*-HA plasmid. However, when YEp-*PUF5* was co-transformed with YCplac33-*CLB1*-HA to cells, the growth was too poor to examine the Clb1-HA protein level. Harboring two plasmids may have caused the poor growth.

3. Clb1 over expression rescues *puf5*Δ *clb2*Δ growth defect, which is also manifested in elongated shaped cells. Can ZDS1 or SSD1 over expression (which perturb similar polarity pathways) also rescue the growth defect of the double knock out?

Since *ZDS1* and *SSD1* strongly suppress the temperature-sensitive growth of the *puf5*Δ mutant, we assume that overexpression of *ZDS1* and *SSD1* also retrieve the growth defect of the *puf5*Δ *clb2*Δ double mutant. However, this effect is likely to be caused by the different regulation from the control of cell growth by Puf5 and Clb2. Therefore, we did not analyze the effect further here.

4. The authors test whether deletion of Lrg1 might have an effect on the genetic interaction between puf5 and clb2 - it is not clear to me why this has been included. The corresponding section in the text should be extended (line 280ff)

According to the reviewer’s comment, we precisely described why we analyzed the effect of *LRG1* deletion on the Results section of the revised manuscript (page 15, line 332-335).

5. To better compare the expression changes in the alpha-factor release experiments, I would suggest to combine figure 8,9 and 10, especially as figure 10 serves as a control for the results in Figure 9.

According to the reviewer’s comment, we considered whether to combine the figures. Consequently, we thought that showing the data in the different figures eases the ordered understanding of the results.

**Reviewer #3**

1. The authors analyzed cell-cycle dependent change of *CLB1* expression using synchronous cultures. They claimed that the peak of *CLB1* mRNA expression was not observed in the *bar1∆ puf5∆* mutant. However, the *CLB1* mRNA level in this mutant apparently showed a low and broad peak around 80–100 min after alpha-factor release in every case (Figs. 8C, 9B, and 10B). If taking some elongated cell-cycle of the *bar1∆ puf5∆* cells expected from level changes of *RNR1* and *SIC1* mRNAs into account, this low, broad and delayed peak seems to be a real peak of *CLB1* mRNA according to cell-cycle progression. Thus, the effect of Puf5, whose deletion causes higher expression of Ixr1, can be interpreted as control of the maximum expression level but not cyclic regulation itself. From the mRNA expression change in Figs. 8–10, one complete cell cycle may not have been covered under these experimental conditions (120 min duration) in the case of the *bar1∆ puf5∆* mutant, so that decrease of the *CLB1* mRNA level upon entering a new G1 phase were not clearly observed in the data. The reviewer recommends to clarify whether Ixr1 affects expression maximum of *CLB1* mRNA or its rhythmicity according to cell-cycle progression.

According to the reviewer’s comment, we examined the cell cycle-dependent expression of *RNR1*, *SIC1*, and *CLB1* until 150 min. These results were added in Figure 9 of the revised manuscript. In the *puf5∆* background, the second peak of the *RNR1* expression, an S phase marker, was observed in the 120 min, and the expression of *SIC1*, a late M phase marker, decreased after the peak at 100-120min. From these data, the cell cycle seems to be completed within the experimental period. While the extent is remarkably small, the *CLB1* expression peaked at 80-100 min. Taking this data into account, we presume that Puf5 and its target Ixr1 affect the maximum expression of the *CLB1*. These data are presented in the Figure 9 of the revised manuscript. We also describe these results in the Results section of the revised manuscript (page 21, lane 516 to page 22, line 532).

2. In a similar line, the authors do not seem to care about difference of the cell-cycle phase (e. g. p. 16, lines 399-401), but both *puf5∆* and *ixr1∆* mutations clearly altered cell-cycle progression, especially recovery from alpha-factor arrest; *puf5∆* showed delayed recovery while *ixr1∆* does earlier recovery. Such difference should be precisely described and discussed.

According to the reviewer’s comment, we described the cell cycle regression in the *puf5∆* mutant and *ixr1∆* the mutant in the Results section of the revised manuscript (page 21, line 519 to page 22, line 526, and page 22, line 543 to page 23, line 551). We also added discussion about the effects of these mutations in the Discussion section of the revised manuscript (page 27, line 661 to page 28, line 669).

3. Finally, if Ixr1 affects cell-cycle dependent rhythmicity of the *CLB1* mRNA level, translational regulation of *IXR1* mRNA may also be important (see Fig. 7D). Did the authors examine cyclic expression of Ixr1 protein and the effect of *puf5* deletion on it?

According to the reviewer’s comment, we examined the Ixr1 protein level throughout the cell cycle in the *bar1∆* cell and the *bar1∆ puf5∆* mutant. These results were added to the Figure 13 and Supplementary Figure 5B-E of the revised manuscript.

Minor comments

1) The authors compared growth phenotypes of several combinations of gene deletions by tetrad analysis in Fig. 3D. But their tetrad sets failed to include an important "positive control" of defective growth, namely a *puf5∆ clb2∆* double mutant. Since the PLOS Genetics data policy does not allow "data not shown," appropriate tetrad sets including *puf5∆ clb2∆* should replace the current tetrad data.

According to the reviewer’s comment, we included additional tetrad analysis data in Figure 3E of the revised manuscript.

2) In Figs. 8–10, visibility of mutant plots is quite poor. The reviewer recommends to use color lines or black dash lines to distinguish data of mutant strains from those of the control. In addition, the authors should clearly indicate what value is set to 1 in the ordinate of the graphs.

According to the reviewer’s comment, we distinguished the data by using different colors in Figure 9-13 of the revised manuscript. We also mentioned about the reference sample whose expression is set to 1 in the revised manuscript.

**About the data availability**

Please be aware that our data availability policy requires that all numerical data underlying graphs or summary statistics are included with the submission, and you will need to provide this upon resubmission if not already present. In addition, we do not permit the inclusion of phrases such as "data not shown" or "unpublished results" in manuscripts. All points should be backed up by data provided with the submission.

According to this comment, we included all numerical data corresponding to graphs in the Supplementary Dataset of the revised manuscript. In addition, we added all the data that were presented as "data not shown" in submission in Supplementary Figure 2 and S5 Table of the revised manuscript.
