# Supplementary material for: The RNA-binding protein Puf5 and the HMGB protein Ixr1 contribute to cell cycle progression through the regulation of cell cycle-specific expression of CLB1 in Saccharomyces cerevisiae
Source: PLoS Genet. 2022 Jul 29;18(7):e1010340. doi: 10.1371/journal.pgen.1010340 (PMC9365169; doi:10.1371/journal.pgen.1010340)
Supplement: Attachment — Submitted filename: Sato_response-to-reviewers_20220713-2.docx [file pgen.1010340.s014.docx]

**Our responses to the reviewers’ comments and changes in the revised manuscript**

Tittle: The RNA-binding protein Puf5 and the HMGB protein Ixr1 contribute to cell cycle progression through the regulation of cell cycle-specific expression of *CLB1* in Saccharomyces cerevisiae

Authors: Megumi Sato, Kaoru Irie, Yasuyuki Suda, Tomoaki Mizuno, Kenji Irie

**Reviewer #3**

1) Although the authors excluded Fkh1 and Fkh2, transcription regulators of CLB1 mRNA, from their primary analyses in p. 17, they picked up these factors again p. 19, especially pointing that parallel contribution of the two genes from the fact that synthetic phenotype of the double deletion. Such development of the point of argument confuses readers.

Furthermore, from p. 19, line 464 to p. 20, line 466, they tried to place Fkh1 and Fkh2 downstream of Ixr1 merely from the fact that IXR1-deletion did not suppress the fkh1 fkh2 double mutant. They failed to appreciate another possibility that Fkh1 and Fkh2 are unrelated to the Ixr1-axis of CLB1 transcriptional regulation.

According to the reviewer’s comment, we edited the sentences about the relationship between Fkh1/Fkh2 and Ixr1 in the re-revised manuscript. We clearly stated the possibility Fkh1 and Fkh2 regulate *CLB1* expression in parallel with Ixr1 (Page 20, lines 468-469 and Page 26, lines 623-625).

Also, as for the point that Fkh1 and Fkh2 appear twice in this story, we edited the sentences about Fkh1/Fkh2 in the re-revised manuscript (Page 17, lines 392-394 and Page 19, lines 460-462).

2) The authors used a word "invariable" to describe expression patterns of IXR1 mRNA and Ixr1-HA protein. However, significant but cell-cycle independent fluctuation of the mRNA amounts is obvious in Fig. 13. They must choose words more precisely.

According to the reviewer’s comment, we edited the sentences about the *IXR1* mRNA and Ixr1-HA protein levels during cell cycle in the re-revised manuscript (Page 23, line 574 – page 24, line 590).

3) The unit of temperature, centigrade (°C), did not appear properly in many parts of the text; for example, p. 8, line 166.

We are sorry that we have not checked the final PDF file carefully. We corrected the unit of temperature in the re-revised manuscript.

4) The mark for "less than or equal to" (≤ ) did not appear properly in p. 13, lines 291 and 293.

We are sorry that we have not checked the final PDF file carefully. We corrected the mark for "less than or equal to" in the re-revised manuscript.
